# A model for an early role of auxin in *Arabidopsis* gynoecium morphogenesis

**DOI:** 10.3389/fpls.2014.00327

**Published:** 2014-07-08

**Authors:** Charles Hawkins, Zhongchi Liu

**Affiliations:** Department of Cell Biology and Molecular Genetics, University of Maryland, College ParkMD, USA

**Keywords:** gynoecium, auxin, ETTIN, abaxial, adaxial

## Abstract

The female reproductive organ of angiosperms, the gynoecium, often consists of the fusion of multiple ovule-bearing carpels. It serves the important function of producing and protecting ovules as well as mediating pollination. The gynoecium has likely contributed to the tremendous success of angiosperms over their 160 million year history. In addition, being a highly complex plant organ, the gynoecium is well suited to serving as a model system for use in the investigation of plant morphogenesis and development. The longstanding model of gynoecium morphogenesis in *Arabidopsis* holds that apically localized auxin biosynthesis in the gynoecium results in an apical to basal gradient of auxin that serves to specify along its length the development of style, ovary, and gynophore in a concentration-dependent manner. This model is based primarily on the observed effects of the auxin transport blocker *N*-1-naphthylphthalamic acid (NPA) as well as analyses of mutants of *Auxin Response Factor (ARF) 3/ETTIN (ETT).* Both NPA treatment and *ett* mutation disrupt gynoecium morphological patterns along the apical–basal axis. More than a decade after the model’s initial proposal, however, the auxin gradient on which the model critically depends remains elusive. Furthermore, multiple observations are inconsistent with such an auxin-gradient model. Chiefly, the timing of gynoecium emergence and patterning occurs at a very early stage when the organ has little-to-no apical–basal dimension. Based on these observations and current models of early leaf patterning, we propose an alternate model for gynoecial patterning. Under this model, the action of auxin is necessary for the early establishment of adaxial–abaxial patterning of the carpel primordium. In this case, the observed gynoecial phenotypes caused by NPA and *ett* are due to the disruption of this early adaxial–abaxial patterning of the carpel primordia. Here we present the case for this model based on recent literature and current models of leaf development.

## THE STRUCTURE OF *Arabidopsis* GYNOECIUM

Angiosperms, plants that produce flowers, are far and away the most diverse division of plants today, with even the most conservative estimates placing the number of known extant species at more than 223,000 (Scotland and Wortley, 2003). In addition to being an incredibly successful group in nature, flowering plants account for the vast majority of plants used and cultivated by humans, both for agricultural and for horticultural purposes. For this reason, there is great promise in the prospect of engineering angiosperm development to increase productivity, fecundity, and survivability. To do that in any systematic way, it is necessary to understand the genetic machinery that drives angiosperm development and that allows these plants to shape themselves into the vast diversity of forms seen in nature.

Evolutionarily, the flower consists of a complex of organs that are derived from leaves growing from a single stem (Coen and Meyerowitz, 1991; Honma and Goto, 2001; Pelaz et al., 2001; Scutt et al., 2006). A complete flower consists of the stem itself, divided into the pedicel and receptacle, and four different types of leaf-derived floral organs arranged in four concentric whorls around the stem. These are, from outermost to innermost: The sepals, which protect the flower; the petals, which serve as a display to attract pollinators; the stamens, which produce pollen; and the carpels, which contain the ovules that later develop into the seeds when they are fertilized. Carpels are of particular interest and significance as they constitute the angiosperms’ defining feature. In many species, the carpels are fused into a single structure called the gynoecium. This structure is of critical economic importance, as it is the source of fruits and of seeds, including nuts, beans, and cereal grains. The interactions of genes and hormones that shape the structure, however, are not completely understood. *Arabidopsis thaliana*, a flowering weed and a model plant, has thus been under intensive investigation to address the underlying molecular mechanisms.

**FIGURE 1:**
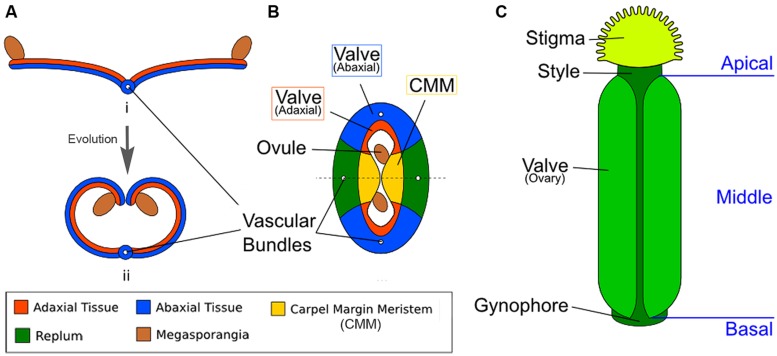
**Diagrams illustrating the homology between modern carpels and ancestral leaves. (A)** Hypothetical evolution of a single plicate carpel based on Scagel (1965). (i) A cross section of an ancestral plant’s spore-bearing leaf (sporophyll), showing megasporangia at the leaf edge. (ii) Over evolutionary time, inward curling of a megasporangia-bearing leaf and subsequent fusion at the leaf margin led to a one-chamber ovary with two rows of megasporangia on the interior (adaxial side). The actual evolutionary path is more complicated and not fully settled. **(B)** The cross section view of the *Arabidopsis gynoecium*, consisting of two fused carpels enclosing two locules. Note the vascular bundles. Although there are four rows of ovules, only two ovules are visible in the cross-section since the rows alternate within each locule. **(C)** A diagram of the *Arabidopsis gynoecium*, showing that it consists of three regions along the basal-to-apical axis. The basal section consists of a short stalk, the gynophore, the middle section is the ovary, and the apex consists of style and stigma.

Like the other floral organs, the carpels are widely thought to represent modified leaves or sporophylls (Balanzá et al., 2006; Scutt et al., 2006; Vialette-Guiraud and Scutt, 2009; Reyes-Olalde et al., 2013). The ancestral carpel is most likely ascidiate, meaning it represents an invagination of a leaf to form a hollow structure sealed by a secretion (Endress and Igersheim, 2000; Endress and Doyle, 2009; Doyle, 2012). There are a number of possibilities as to how exactly this occurred, including curled leaf borne on axillary branch or curled leaflets borne along the rachis of a compound leaf (Doyle, 2012). Examples of ascidiate carpels can be found in the basal extant angiosperms such as *Amborella* and water lilies. Most “higher” angiosperms, however, including most monocots and eudicots (*Arabidopsis* among them), instead possess plicate carpels (Endress and Doyle, 2009; Doyle, 2012). Rather than being an invagination of the leaf, the plicate carpel is curled or folded along its length into a tube-like or book-like shape, enclosing the ovules within (**Figure 1A**). This type of structure appears to have evolved by elongation of the apical end of the primitive ascidiate carpel. In angiosperms, irrespective of carpel type, the ovule-bearing surface is strictly adaxial (Doyle, 2012).

In *Arabidopsis*, two carpels are fused congenitally to form the gynoecium (Sattler, 1973; **Figure 1B**), and each carpel is homologous to an ancestral spore-bearing leaf (sporophyll; compare **Figure 1A** with **Figure 1B**). The adaxial tissues near the margins of the fused carpels are meristematic and are thus called the carpel margin meristem (CMM; **Figure 1B**). The CMM is responsible for generating the placenta, ovules, septum, transmitting tract, style, and stigma; these tissues are critical for the reproductive competence of the gynoecium (Wynn et al., 2011; Reyes-Olalde et al., 2013). From the base to the apex of the gynoecium are three morphologically distinct regions (**Figure 1C**). The basal-most region is the gynophore, a short stalk that connects the rest of the gynoecium to the flower. The apical-most region of the gynoecium consists of the style and stigma. In the middle of the gynoecium is the ovary; a cross section of the ovary (**Figure 1B**) shows two valves (also called ovary valves or carpel valves) separated externally by the replum and internally by a septum, dividing the interior into two locules. Each locule protects two rows of ovules initiated along the carpel edges from the CMM.

The homology between carpels and leaf-like lateral organs extends to the resemblance of carpel valves to leaf blades (lamina) and the CMM to the leaf margins. In certain angiosperm species such as *Kalanchoe daigremontiana,* also known as “mother of thousands,” leaf margins produce plantlets and express the meristem marker gene *SHOOT MERISTEMLESS* (*STM*) in a small group of leaf margin cells that were initiating plantlets (Garcês et al., 2007), much like the *STM*-expressing placenta along the *Arabidopsis* carpel margins (Long et al., 1996). The possibility of conserved molecular mechanisms that specify the basic organ plan of the leaf and carpel draws support from several prior observations: Firstly, *N*-1-naphthylphthalamic acid (NPA) treatment causes the formation of both needle-like leaves without a lamina and of stalk-like gynoecia without valves (Okada et al., 1991). Further, NPA treated young leaves showed increased density of veins along their margins and multiple parallel midveins, much like NPA-treated gynoecia where the veins linking the gynoecium to the receptacle are increased in number (Nemhauser et al., 2000). Secondly, when one manipulates the expression of A, B, C, and E-class floral homeotic genes, floral organs can be turned into leaves or vice versa (reviewed in Goto et al., 2001). Thirdly, single sepals can be readily turned into single, free carpels, such as in *Arabidopsis ap2-2* mutants (Bowman et al., 1989).

## AUXIN REGULATES GYNOECIUM DEVELOPMENT

Of critical importance to the development of the plant is auxin, a family of hormones of which the most common is indole-3-acetic acid (IAA). This tryptophan-derived chemical is needed for many different processes in the plant, including lateral organ initiation and morphogenesis, phototropism, lateral root initiation, xylem formation, and apical dominance (Arteca, 1996; Benková et al., 2003; Friml, 2003). Auxin was the first plant hormone to be identified and has classically been characterized as a hormone synthesized in growing apices and transported down toward the roots.

### AUXIN BIOSYNTHESIS

The IAA biosynthetic pathway begins with tryptophan or a tryptophan precursor (Bartel, 1997; Ljung, 2013). Recent reports suggest that auxin biosynthesis in plants involves only a two-step pathway, in which *TRYPTOPHAN AMINOTRANSFERASE OF ARABIDOPSIS1 (TAA1)* and its four homologs *TAR1-4* convert tryptophan to indole-3-propionic acid (IPA). Members of the *YUCCA (YUC*) family of flavin monooxygenases then catalyze the conversion of IPA to auxin (Mashiguchi et al., 2011; Stepanova et al., 2011; Won et al., 2011; Zhao, 2012).

**FIGURE 2:**
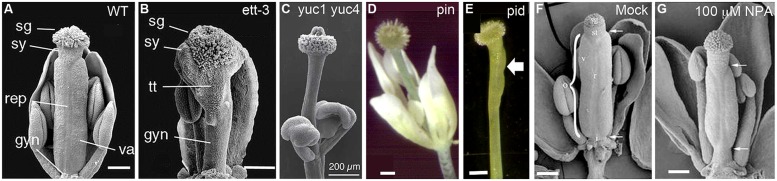
**Gynoecium phenotypes of mutants defective in auxin biosynthesis, transport, or signaling. (A)** Wild-type gynoecium at stage 12 with the parts labeled as stigma (sg), style (sy), replum (rep), valves (va), and gynophore (gyn). **(B)** ett-3 gynoecium at stage 12, showing an elongated gynophore, a diminished valve pushed toward the apex, and expanded stigma, style, and transmitting track (tt) tissue. **(C)** Gynoecium of a yuc1-1 yuc4-1 double mutant, showing the complete absence of ovary valve and an enlarged apical stigma. **(D)** A weak pin mutant showing a gynoecium without any ovary valve tissue. **(E)** A pid gynoecium with one small ovary valve (arrow). **(F,G)** NPA-treated wild type *Arabidopsis* gynoecium. The apical and basal boundaries of the ovary are marked by a pair of arrows. The various tissues are indicated with letters: ovary (o), replum (r), valve (v), style (st), and stigma (sg). Images are reproduced from Heisler et al. (2001; **A,B**), Cheng et al. (2006; **C**); Roeder and Yanofsky (2006; **D,E**), and Nemhauser et al. (2000; **F,G**) with permissions from Copyright Clearance Center or Creative Commons Attribution-Non-Commercial 4.0 International License. Scale bars: 200 μm **(A–C)**; 250 μm **(D,E)**; 165 μm **(F)** and 140 μm **(G)**.

Analyses of the expression and mutant phenotypes of auxin biosynthesis genes indicate that localized synthesis of auxin is critical to proper gynoecium morphogenesis. Among the 10 *YUC*-family genes, *YUC1* and *YUC4* appear to play important roles in gynoecium development (Cheng et al., 2006) as double *yuc1 yuc4* mutants show a stalk-like gynoecium (**Figures 2A,C**), completely missing the ovary valves. *In situ* hybridization and promoter-*GUS* (β-glucuronidase) fusions have revealed that both *YUC1* and *YUC4* are expressed in inflorescence apices and young floral primordia. Most interestingly, *YUC1* and *YUC4* are expressed at the base of young floral organs including carpel primordia (Cheng et al., 2006). This specific expression pattern at the base of emerging floral organs is likely critical to proper floral organ initiation and apical–basal patterning (see later sections). In older flowers, *YUC4* expression is concentrated at the apical tip of carpels, stamens, and sepals (Cheng et al., 2006) and may be involved in later proper differentiation of floral organs.

Likewise, double mutants of TAA1/TAR family genes exhibit stalk-like gynoecia similar to those of *yuc1 yuc4* double mutants (Stepanova et al., 2008). The TAA1-GFP protein is localized in a few cells located at the apex (L1 layer) of young floral primordia as early as floral stage 2. This localized expression continues to floral stage 4, when a few epidermal cells at the central dome of the carpel primordium express TAA1. Since floral stage 4 is when carpel primordia emerge, this localized TAA1 expression may be involved in the apical–basal patterning of the gynoecium. Later, at floral stages 5–9, TAA1-GFP is prominently expressed in the medial ridge region of the gynoecium; this later stage expression maybe relevant to the development of marginal tissues including ovules, styles, and stigma. Based on localized and specific expression patterns of TAA1/TAR, Stepanova et al. (2008) suggested that auxin is synthesized in different regions at different developmental times and that localized auxin biosynthesis may represent a mechanism redundant to auxin transport in ensuring that robust local auxin maxima are able to form.

### AUXIN SIGNALING

Auxin signaling consists of a system of the TIR/AFB family of receptors, the IAA family of repressors, and the *ARF* family of transcription factors. ARFs contain a DNA binding domain but most require homodimerization to bind DNA (Ulmasov et al., 1999). IAA-family repressor proteins bind to ARFs and competitively inhibit their ability to homodimerize. The TIR/AFB family of auxin receptors, when bound by auxin, induces the ubiquitination and degradation of the IAA repressors, thus freeing the ARFs to bind DNA. This may result in transcriptional activation or repression of target genes, depending on the co-factors bound to the ARF (Dharmasiri et al., 2005; Kepinski and Leyser, 2005; Mockaitis and Estelle, 2008; Calderón Villalobos et al., 2012). AUXIN BINDING PROTEIN1 (ABP1) represents a second type of auxin receptor, which acts as part of a system of rapid and local auxin responses on the plasma membrane (Dahlke et al., 2010; Xu et al., 2010; Effendi and Scherer, 2011; Shi and Yang, 2011; Craddock et al., 2012). The plasma membrane localized TMK1 receptor-like kinase was recently found to physically associate with ABP1 at the cell surface to regulate ROP GTPase signaling in response to auxin (Xu et al., 2014). In addition, ABP1 also acts to negatively regulate the SCF (TIR/AFB)-mediated auxin signaling pathway (Tromas et al., 2013).

*ETTIN* (*ETT*), also known as *ARF3*, is a member of the ARF family. Its closest in-paralog is *ARF4*, from which it appears to have split early in angiosperm evolution (Finet et al., 2010). *ETT* and *ARF4* are also expressed in the abaxial domain of leaves and floral organs, where they are believed to function as abaxialization factors in lateral organ development (Sessions et al., 1997; Pekker et al., 2005; Hunter et al., 2006). In the gynoecium, *ett* mutants show diminished or absent carpel valve tissue and an expansion of stigma, stylar, and basal gynophore (**Figure 2B**; Sessions and Zambryski, 1995; Sessions, 1997; Sessions et al., 1997; Heisler et al., 2001). The severe gynoecium phenotype of *ett* provided one of the earliest clues pointing to auxin as a critical regulator of gynoecium morphogenesis.

### AUXIN TRANSPORT

Auxin travels through the plant via a cell-to-cell, “bucket brigade” style of transport. According to the chemiosmotic model, first proposed by Rubery and Sheldrake (1974), the acidic environment of the extracellular space (the apoplast) protonates the auxin, allowing IAA to diffuse across the plasma membrane into adjacent cells. Once inside a cell, it is exposed to a more alkaline pH and becomes deprotonated. The resulting anionic IAA^-^ is unable to cross the lipid bilayer without the help of eﬄux carriers. There are two different families of eﬄux transport proteins. The *PIN-FORMED* (*PIN)* family of eﬄux carriers is localized to a particular pole of the cell, exporting IAA selectively in the direction corresponding to PIN’s localization (Wiśniewska et al., 2006; Löfke et al., 2013). The ATP Binding Cassette B (ABCB) transporters represent the second type of auxin eﬄux transporters. ABCB and PIN can independently as well as coordinately transport auxin (Titapiwatanakun and Murphy, 2009; Peer et al., 2011). Distinct modes of directional auxin transport operate in different developmental contexts. “Up-the-gradient” PIN1-based transport generates auxin maxima at lateral organ initiation sites, while “with-the-flux” PIN1 polarization operates in leaf midvein patterning (Bayer et al., 2009).

A third class of auxin transport proteins is the AUX1/LAX family of auxin uptake symporters. Though IAA is believed to be capable of entering a cell from the apoplast by passing through the membrane on its own (Rubery and Sheldrake, 1974), these auxin uptake symporters are still necessary for a number of developmental processes due to their ability to create sinks for auxin to flow into (reviewed in Titapiwatanakun and Murphy, 2009; Peer et al., 2011). In addition, AUX1 was proposed to play a role in restricting auxin to the epidermis of vegetative meristems by counter-acting the loss of auxin caused by diffusion into the meristem inner layers (Reinhardt et al., 2003).

Strong null mutants of *PIN1* produce no lateral organs or axillary shoots, resulting in the bare, pin-like shoot that gives the mutants their name (Okada et al., 1991; Gälweiler et al., 1998; Palme and Gälweiler, 1999; Benková et al., 2003). In weak *pin* mutants, lateral organs can develop but the gynoecium is often valveless and topped with stigmatic tissues, which is reminiscent of the abnormal gynoecium of auxin biosynthesis mutants described above (compare **Figures 2C,D**). *PINOID (PID)*, an AGC3-type protein kinase, acts to phosphorylate PIN to regulate PIN’s polar localization in the cell (Friml et al., 2004; Huang et al., 2010). Interestingly a similar gynoecial phenotype was observed in *pid* mutants (**Figure 2E**; Bennett et al., 1995; Benjamins et al., 2001). The action of *PIN* proteins in transporting auxin may be blocked via the application of NPA. Application of NPA to wild type *Arabidopsis* mimics *pin* mutant phenotypes (Okada et al., 1991; Nemhauser et al., 2000) with pin-like shoots as well as abnormal gynoecia without any valve or with reduced valves (**Figures 2F,G**). Taken together, while severe disruption of polar auxin transport abolishes all lateral organ initiation and hence results in the formation of pin-like shoots, milder disruption of polar auxin transport allows lateral organ initiation but blocks proper lateral organ morphogenesis, resulting in stalk-like gynoecia (**Figures 2D,E**). The weaker *pin* and *pid* mutant phenotypes provide strong evidence that polar auxin transport is critical for gynoecium morphogenesis.

## THE NEMHAUSER MODEL OF GYNOECIAL PATTERNING

Multiple lines of evidence strongly indicate that the action of auxin is critical for proper development and apical to basal patterning of the gynoecium. Mutants of biosynthesis (*yuc* or *taa/tar*) and transport (*pin* and *pid*) genes show the strongest gynoecium phenotype, a phenotype that is nearly identical between them: their valveless gynoecium is basically a thin and round stalk topped with stigmatic tissues (**Figures 2C–E**). Application of the polar auxin transport inhibitor NPA shows a similar but weaker phenotype with reduced ovary valves (**Figures 2F,G**). While mutations in the auxin signaling gene *ett/arf3* cause a similar effect to those of auxin biosynthesis (*yuc/taa/tar*) or transport (*pin/pid*) in reducing ovary valve, *ett/arf3* mutants appeared to exhibit more expanded stigma and stylar tissues (**Figure 2B**).

**FIGURE 3:**
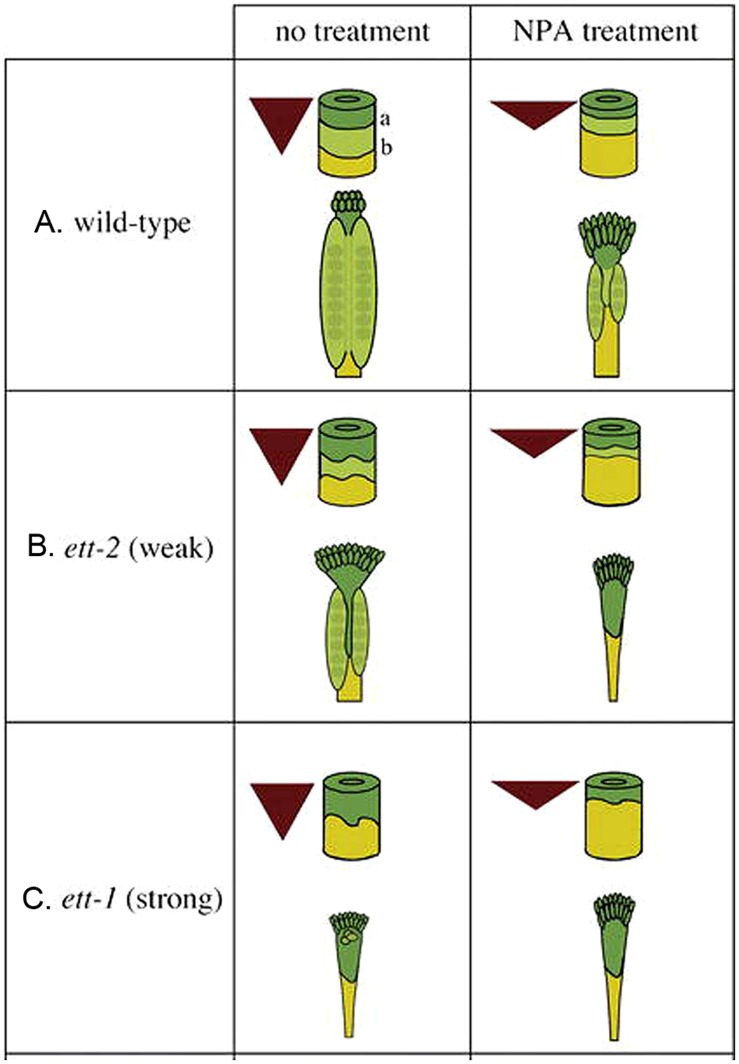
**The auxin gradient model.** Auxin is produced at the apex and transported toward the base, creating a morphogenic gradient that provides positional information, which is interpreted in part by *ETT* to specify ovary valve. The triangle represents the auxin gradient within the gynoecium. The cylinder represents the gynoecium with border marked “a” between the style (dark green) and ovary (light green) and border marked “b” between the ovary and gynophore (yellow). **(A)** Wild-type gynoecia with and without NPA treatment. **(B)** Weak *ett-2* mutants with a mild phenotype (left); the phenotype is significantly enhanced when *ett-2* mutants were treated with NPA (right). **(C)** Strong *ett-1* mutants with a strong phenotype with or without NPA treatment. The figure is reproduced from Nemhauser et al. (2000) with permission from Copyright Clearance Center.

Based on the phenotype of *ett/arf3* and the effect of NPA treatment on wild type and *ett/arf3* gynoecia, Nemhauser et al. (2000) proposed a model wherein auxin biosynthesized locally at the apex of the gynoecium is transported basipetally, resulting in a gradient of auxin concentration with a maximum at the apex, mid-range level in the middle, and a minimum at the base (**Figure 3A**). The high auxin level at the apex specifies stigma/style, while the mid-range level promotes valve formation. At the base when auxin level is low, gynophore develops. *ETT* is partly responsible for interpreting this gradient, and promotes the formation of valve tissue in the middle region of gynoecium where there is a mid-range level of auxin. Under this model, when the gynoecium is exposed to NPA, the auxin produced at the apex is not transported down as readily, resulting in a steeper and up-shifted gradient (**Figures 3A–C**). This results in the observed phenotype of a smaller amount of valve tissue being formed near the apex of the gynoecium and a “bushier” stigma, which could be explained under this model by pooling and accumulating a higher level of apically synthesized auxin at the gynoecium apex. Because of the shift of auxin gradient toward the apex, the basal region, the gynophore, is expanded (**Figures 3A–C**). Mutants of *ETT*, under this model, show a similar phenotype because the job of *ETT* is to interpret the mid-range auxin gradient in the middle segment of the gynoecium to promote valve formation. In the absence of *ETT*, therefore, the auxin gradient is invisible to the plant, and valve fails to form (**Figure 3C**).

This model was reasonably consistent with the data available at the time. Since then, however, additional information has emerged. The auxin biosynthesis gene *YUC4* is expressed (among other places) in a small region at the tip of multiple lateral organs, including cotyledons, and stamens. However, it does so largely when the organs are close to maturity (Cheng et al., 2006). In the gynoecium, the apical *YUC4* expression is not visible until after the gynoecial apical-to-basal patterning is largely determined (after stage 7–8; Cheng et al., 2006) and thus is not likely to be responsible for the initial pattern formation of the gynoecium. At earlier stages of floral meristem development (stages 3–7; staging based on Smyth et al., 1990), *YUC4* as well as *YUC1* are expressed at the bases of young floral organ primordia, including the base of young gynoecia. In light of the timing and the dramatic gynoecium phenotype of *yuc1 yuc4* double mutants (**Figure 2C**), the early expression pattern around young floral primordia maybe more relevant to gynoecial apical-to-basal patterning than the later-stage *YUC4* expression at the apex. Further, if auxin is made at the apex and responsible for stigma formation, we would expect to see a reduced or diminished stigmatic tissue in *yuc1 yuc4* double mutants. However, *yuc1 yuc4* double mutants as well as *taa/tar* double mutants produce heads of stigmatic tissue even larger than wild type and their phenotypes are little different from those of plants that fail to transport auxin and therefore supposedly pool the auxin at the apex due to a lack of downward transport (compare **Figure 2C** with **Figures 2D,E**; Cheng et al., 2006; Stepanova et al., 2008).

Various attempts have been made to visualize the proposed auxin gradient using the *DR5* reporter. DR5 consists of tandem direct repeats of an 11-bp auxin-responsive element and, when used to drive a reporter gene, serves to report local auxin response (Ulmasov et al., 1997). Larsson et al. (2013) examined auxin distribution during early stage gynoecium development (about stage 7) using the *DR5rev::GFP* reporter. Two weak foci were detected at the apical tips of stage 7 flowers. At later stages (about stage 8), *DR5rev::GFP* expression was expanded into four foci (both medial and lateral domains) and in the pro-vasculature. Throughout the development, no gradient was observed. Other experimental work has also shown localization of auxin only to the apex of gynoecia in flowers at stage 6 or older, without showing a gradient along the apical-to-basal axis at any stage (Benková et al., 2003; Girin et al., 2011; Grieneisen et al., 2013). These data do not support the auxin gradient model.

Finally, the auxin gradient model proposed that the auxin is transported in a basipetal direction. Yet studies of the polar localization of auxin eﬄux carrier PIN1 show accumulation in the apical side of the replum cells (Sorefan et al., 2009; Grieneisen et al., 2013), indicating upward transport.

Fourteen years after the proposal of the auxin gradient model, accumulating new data suggest that this model, while highly attractive at the time it was proposed, should be revised or re-evaluated. Alternative models that better interpret and incorporate these new observations should be proposed.

## OTHER ALTERNATIVE MODELS

Prior to the Nemhauser’s auxin gradient model, Sessions (1997) proposed a “boundary” model, in which *ETT* was proposed to regulate the two boundary lines that trisect the gynoecium into three regions, with one boundary (the apical line) dividing the ovary from the stylar tissues and the second boundary (the basal line) dividing the gynophore from the ovary above it. Sessions (1997) further proposed that the two boundaries are set as early as stage 6 of flower development, when the effects of *ett* begin to be observed. Based on this model, the effect of *ett* was interpreted as simultaneously lowering the apical boundary line and raising the basal boundary line. These two lines are also proposed in the Nemhauser model (**Figure 3**), which was built upon Sessions’ “boundary” model. Since the molecular identify of *ETT* as an ARF was not published at the time when the “boundary” model was proposed, the connection to auxin was not proposed. Although Sessions (1997) mentioned an adaxial/abaxial boundary located at the distal tip of the carpel primordia, *ETT* was not proposed to regulate the adaxial/abaxial boundary.

Recently, Larsson et al. (2013), unable to detect an auxin gradient along the apical-to-basal axis of early stage gynoecium using the *DR5rev:GFP* reporter described above, pointed out that their data did not strongly support the Nemhauser gradient model. In addition, Larsson et al. (2013) noted the fact that auxin biosynthesis genes are expressed in regions not limited to the gynoecium apex as another inconsistency with the Nemhauser gradient model. They then proposed several alternative ideas/models. One was the proposal of an abaxial domain KANADI (KAN)–ETT complex that regulates PIN activity and localization during positional axis determination in gynoecia. This idea directly links AD/AB polarity with auxin in the determination of the apical-to-basal axis of gynoecia and is similar to what is being proposed below. Another idea put forth by Larsson et al. (2013) was the differential sensitivity or response of the lateral vs. medial tissues of gynoecium to auxin polar transport inhibitors.

## LESSONS FROM LEAF MORPHOGENESIS

Auxin has long been known to play a role in leaf initiation. Auxin is observed to pool in small areas (maxima) on the shoot apical meristem, and the appearance of such an auxin maximum presages the formation of each lateral organ primordium (Reinhardt et al., 2000, 2003; Benková et al., 2003; Heisler et al., 2005; Scarpella et al., 2006; Smith et al., 2006). An auxin maximum in the L1 layer of the meristem is the earliest mark of a new lateral organ primordium. The formation of such auxin maxima correlates with localization of the membrane-associated auxin eﬄux carrier PIN1, in each epidermal cell, to the side of the cell that faces toward the neighbor with a higher auxin concentration. This “up-the-gradient” transport helps to amplify the localized concentration of auxin. Heisler et al. (2005) showed *pPIN1::PIN-GFP* localization in the L1 layer toward incipient primordia starting at incipient primordium stage 3 (I3; from youngest to oldest, the stages are I3, I2, I1, budding-primordium1 (P1, P2, etc.). The signal intensity of the polarized PIN-GFP toward the auxin maxima increased steadily until primordial stage P1. The PIN1-GFP in the adaxial domain of lateral organ primordia then showed a brief reversal of transport, switching from being directed toward the primordium to being directed away from the primordium. These two waves of auxin transport suggest that auxin may act twice in lateral organ development, first in organ primordium initiation and then possibly in organ growth. If so, the timing and specific context of auxin flow may affect different processes of organ development.

**FIGURE 4:**
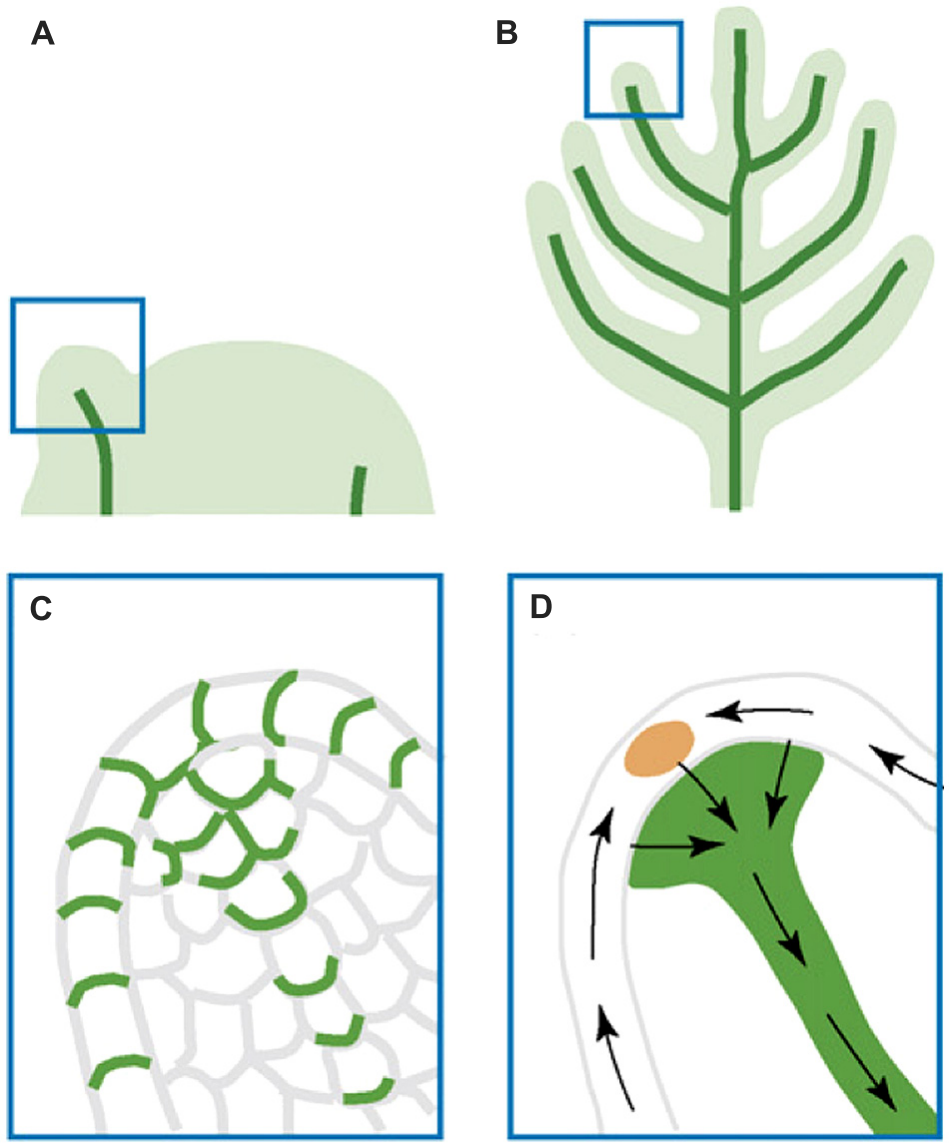
**Illustration of auxin transport during leaf and lateral organ initiation. (A)** Leaf primordial initiation. **(B)** Lateral organ initiation. **(C)** A zoom-in diagram of the leaf primordium tip showing PIN:GFP (green) polar localization that indicates auxin transport routes. **(D)** Inferred auxin transport routes (black arrows) based on PIN:GFP localization. The epidermal convergence of two counter-oriented auxin flows results in a change of auxin transport direction toward the internal base of the primordium. This internal flow is responsible for the formation of the midvein. The figure is reproduced from Berleth et al. (2007) with permission from Copyright Clearance Center.

The function of auxin maxima and polar auxin transport in lateral organ initiation and growth was demonstrated by examining *pin* mutants, where auxin maxima as well as lateral organ formation were absent. Further, application of auxin to the peripheral zone of the meristem induces lateral organ formation (Reinhardt et al., 2000, 2003; Smith et al., 2006). However, Smith et al. (2006) showed that short-term NPA treatment failed to abolish the auxin maxima, suggesting the presence of additional mechanisms that help redistribute auxin within the epidermis of the shoot apical meristem. On reaching their convergence point, the auxin flows switch the direction and go basipetally toward the roots (**Figures 4A–D**; Berleth et al., 2007). The internal auxin flows are responsible for the leaf midvein formation and utilize the “with-the-flux” transport mode (Bayer et al., 2009).

Soon after a leaf primordium is initiated, one of the first signs of patterning appears in the specification of the adaxial (upper; AD) and abaxial (lower; AB) halves of the leaf. This early patterning is believed to happen in response to a signal generated at the apex or shoot apical meristem (Sussex, 1951; reviewed in Husbands et al., 2009). If the path from shoot apex to primordium is blocked, such as by a cut made directly above the incipient primordium, the adaxial–abaxial patterning of the leaf will be disrupted. The identity of this signal is still unknown but auxin remains a possibility (Husbands et al., 2009).

The AD and AB domains not only exhibit characteristic cell morphology but also express cohorts of domain-specific genes (reviewed in Kidner and Timmermans, 2007; Liu et al., 2012). These gene cohorts, generally mutually repressive, will remain associated with the AD and AB sides of the leaf as they develop. Therefore, the earliest differentiation of the AD and AB domains in lateral organ primordia can be detected by examining AD- and AB-specific marker genes. As early as stage I1, the adaxial marker *REVOLUTA (REV; pREV::REV-VENUS)* was found to be visibly expressed in the adaxial domain of incipient primordia while the abaxial marker gene *FILAMENTOUS FLOWER (FIL; pFIL::DsRED-N7*) was expressed in the abaxial domain (Heisler et al., 2005). Further, *pPIN1::PIN1-GFP* expression was found to mark the boundary between AD and AB domains marked, respectively, by *pREV::REV-VENUS* and *FIL::dsRED-N7* (Heisler et al., 2005). Based on these results, Heisler et al. (2005) proposed that the auxin transport route plays a role in positioning the boundary between adaxial and abaxial cells. Barton (2010) also noted that the AD/AB boundary in a primordium coincides with the point in the primordium on which the epidermal auxin flows from opposite directions converge. If causal, this would indicate that a specific role of auxin transport is to establish the AD/AB boundary in incipient organ primordia.

Proper specification of the AD/AB domains is critical for proper leaf development because it generates the AD/AB boundary and the juxtaposition of AD and AB domain is essential for leaf blade formation (Waites and Hudson, 1995). Many of these AD/AB polarity genes are required for the leaf to grow a blade (lamina), and disruption of one or more of them often creates needle-like structures, with the lamina absent or severely reduced. Examples of this include single mutants of the adaxialization factor *PHANTASTICA* in *A. majus* (Waites and Hudson, 1995), double or triple mutants of the abaxialization factor family *KAN* (Eshed et al., 2004; Pekker et al., 2005), mutants of the HD-ZIPIII adaxially localized proteins (McConnell and Barton, 1998; Emery et al., 2003), and mutants of YABBY genes (Stahle et al., 2009; Sarojam et al., 2010).

*ETT/ARF3* and its paralog *ARF4*, both auxin signaling components, have been suggested as the essential intermediaries for the gradual establishment of abaxial identity in lateral organs initiated by *KAN*. *KAN* encodes a GARP transcription factor and plays a key role in the abaxial identity specification of leaves, carpels, embryos, and vasculature (Eshed et al., 2001, 2004; Kerstetter et al., 2001; Ilegems et al., 2010). Since *KAN* does not regulate *ETT*/*ARF4* transcription, and over-expression of *ETT* or *ARF4* cannot rescue *kan1 kan2* double mutants, they are thought to act cooperatively (Pekker et al., 2005). Interestingly, ETT has been found to physically interact with a KAN family protein, ATS/KAN4 (Kelley et al., 2012). This ETT–KAN complex likely acts in different developmental contexts, embryogenesis, integument development, and leaf lamina growth, by promoting abaxial fate and repressing adaxial fate (Kelley et al., 2012).

Recently it was shown that KAN1 and the adaxial HD-ZIPIII factor, REV, oppositely regulate genes in auxin biosynthesis, transport, and signaling (Merelo et al., 2013; Huang et al., 2014). KAN was shown to regulate PIN1 expression and localization during embryo as well as vascular development (Izhaki and Bowman, 2007; Ilegems et al., 2010). Additionally, the AS1–AS2 nuclear protein complex involved in leaf AD/AB polarity specification was recently shown to directly and negatively regulate *ETT* (Iwasaki et al., 2013). These experiments indicate that proper AD/AB polarity establishment and maintenance in leaves critically depend on proper regulation of auxin synthesis, transport, and signaling. Thus, dynamic auxin regulation and AD/AB polarity specification and maintenance appear to regulate each other in a feedback loop in different tissue and developmental contexts. Any disruption in auxin synthesis, transport, and signaling will affect AD/AB polarity and vice versa.

## A NEW MODEL: THE EARLY ACTION MODEL OF AUXIN ON GYNOECIUM PATTERNING

The evolutionary derivation of floral organs from leaf-like lateral organs suggests that the basic molecular tenets of the regulation of lateral organ polarity may be conserved. Indeed, carpels, like leaves, express members of the same gene families that control leaf AB/AD polarity. *ETT* and *ARF4* are clearly involved in carpel development and show abaxial domain-specific expression around the outer side of the tube of the developing gynoecium, the side that is equivalent to the underside of the leaf (Pekker et al., 2005). Similarly, the expression of class III HD-ZIP adaxialization factor *PHABULOSA* (*PHB*) and the abaxialization factor *YABBY1* (*YAB1*) are detected in the carpels in an equivalent configuration to that of members of their respective families found in the leaf (Franks et al., 2006; Nole-Wilson et al., 2010).

If an individual carpel primordium develops in an analogous manner to that of a leaf primordium, the AD/AB boundary of the carpels should be set very early in their development, at the incipient carpel primordium stage (approximately at floral stage 3–4). Further, auxin should have a major role to play at this stage in specifying the initial AD/AB boundary. The expression of the *YUC1* and *YUC4* genes suggests that auxin production is likely localized to the base of individual floral organ primordia at the very beginning of the primordial initiation (Cheng et al., 2006); this local auxin production and subsequent transport may contribute, at least partly, to the establishment of the AD/AB boundary in developing carpel primordia. As suggested by Stepanova et al. (2008), localized auxin biosynthesis and transport may represent a mechanism redundant to the transport of auxin from elsewhere to ensure robust local auxin maxima at the organ primordia. The site of auxin maximum at the incipient carpel primordium may set the sharp AD/AB boundary, as has been proposed for leaves and lateral organs (Heisler et al., 2005; Barton, 2010).

**FIGURE 5:**
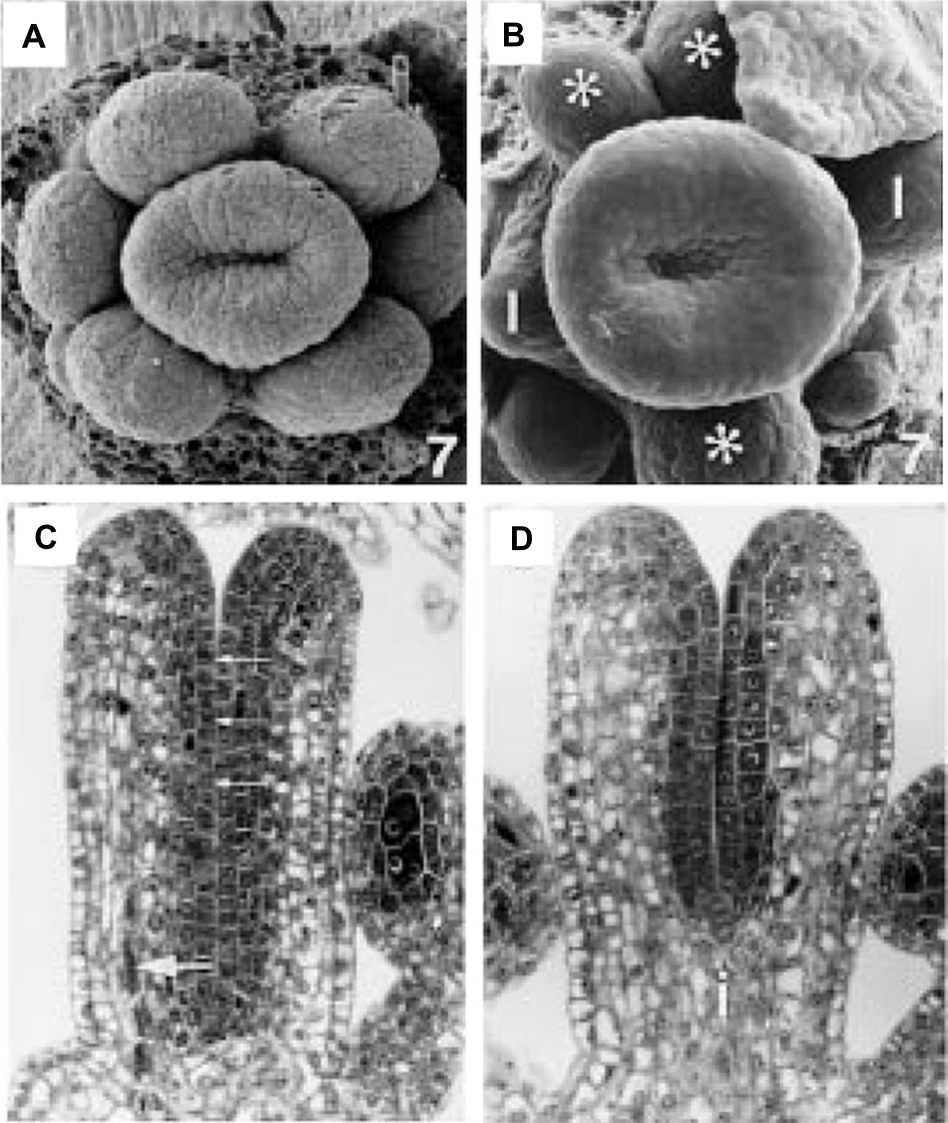
**Early stage wild type and *ett-1* gynoecium development. (A)** Stage 7 wild type floral meristem showing upward growth of the gynoecial tube. **(B)** Stage 7 *ett-1* floral meristem showing a shallower gynoeciual tube. Aberrant stamen is marked with *. Scale bar is 22 μM **(A)** and 30 μm **(B)**, respectively. **(C)** Section of the medial plane of a stage 8 wild type gynoecium showing inner surface (small arrows) and medial vascular bundle (large arrow). **(D)** Section in the medial plane of a stage 8 *ett-1* gynoecium showing a shorter tube. The basal gynophore (i) is more prominent. Images reproduced from Sessions (1997) with permission from *American Journal of Botany*.

**FIGURE 6:**
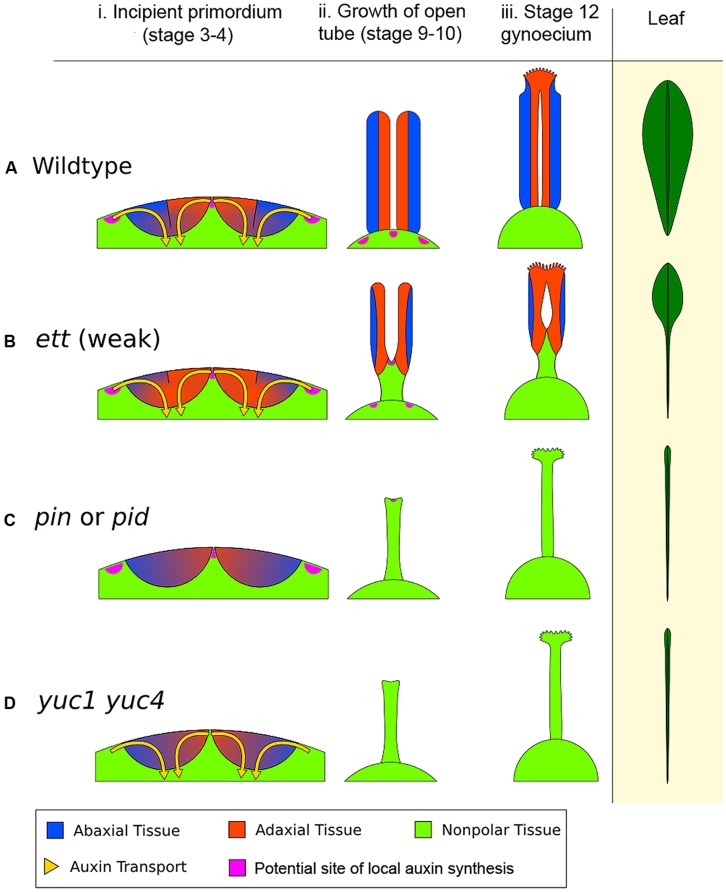
**The early action model of gynoecium patterning. (A)** Wild type (WT) gynoecium development. The diagram in (i) depicts a young floral meristem giving rise to the two incipient carpel primordia, viewed as an enlarged longitudinal section of the floral meristem apex. In WT, opposing auxin flows (indicated by the yellow arrows) converge on the epidermal center of each carpel primordium. The convergence site likely marks the AD/AB boundary, shown as a black line between blue (AB) and orange (AD) domains. The sharp AD/AB boundary ensures upward growth of carpel tube, forming a long tube with AD domain facing interior (ii). Later the cylindrical tube differentiates into stigma/style at the apex and barely visible gynophore at the base (iii). The phenotypic analogy to a normal *Arabidopsis* leaf with lamina along its entire length is shown on the right. **(B)** In a weak *ett* mutant (*ett-2*), abaxial identity is compromised (but not eliminated entirely), resulting in partial adaxialization of the carpel primordia indicated by expansion of orange color (AD) area (i). As a result, there is diminishing AD/AB boundary, indicated by a shorter boundary line (i). Consequently, only a small area of the carpel primordium near the primordial apex has a clear AD/AB boundary. This shorter (or fuzzier) AD/AB boundary results in limited upward growth and hence a shorter (shallower) tube (ii), and subsequently a reduced ovary valve (iii). This phenotypically resembles leaf polarity mutants (such as double mutants of *KAN*) with a diminished lamina pushed to the leaf tip. **(C)** In auxin polar transport mutants such as in *pin* or *pid* mutants, the two counter-oriented auxin flows are compromised, resulting in failure to form a sharp AD/AB boundary as well as a lack of clear AD or AB identity, which is indicated by mixed blue-orange color in the primordia (i). Since the AD/AB boundary is required for valve formation, a lack of the AD/AB boundary resulted in only radialized gynophore (ii and iii), which exhibits no AD/AB polarity. **(D)** In auxin biosynthesis mutants such as in the *yuc1 yuc4* double mutants, a lack of local auxin biosynthesis, and hence a reduced auxin flow, results in little or no AD and AB identity being formed and no AD/AB boundary being established, as indicated by the mixed blue-orange color (i). Without the AD and AB polarity boundary, there is little to no carpel valve growth (ii, iii), analogous to a leaf without lamina (Waites and Hudson, 1995), shown on the right diagram. The pink patches highlight putative local auxin synthesis sites based on Cheng et al. (2006). The medial region expression of *TAA1* in gynoecium at floral stages 5–9 (Stepanova et al., 2008) is not shown.

Based on the ideas put forward by Larsson et al. (2013) linking AD/AB polarity to auxin in the determination of the apical-to-basal axis of gynoecia, we further propose that proper AD/AB polarity establishment and boundary juxtaposition in carpels is necessary for the upward growth of the carpel valve, analogous to the requirement of AD/AB boundary juxtaposition in leaf lamina formation. The valveless gynoecia in auxin pathway mutants are therefore much like the bladeless leaves of polarity mutants. Since the two carpels are congenitally fused, their primordia rise as a circular ring (**Figure 5A**; Sessions, 1997). We propose that the AD/AB boundary likely resides at the apical ridge of the ring. The close juxtaposition of AD and AB domains on either side of this boundary causes the ring ridge to grow vertically as a long hollow tube with adaxial tissues facing inward (**Figure 5C**). However, at the base of the gynoecium primordium, the AD/AB boundary is diffuse, resulting in the base of the primordium developing into a single radially symmetric and non-hollow gynophore. If the AD/AB boundary is disrupted, for example in *ett* mutants, the upward growth of the ring ridge fails to occur, or only occurs to limited extent resulting in a shallower tube (**Figures 5B,D**). The elongation of the gynophore may be regulated by a separate mechanism related to the proximal–distal growth similar to the elongation of needle-like leaves in polarity mutants. **Figure 6** depicts the early action model in wild type and different auxin pathway mutants. In wild type (**Figure 6A**), each incipient carpel primordium is divided into AD and AB domains at the site of convergence of the two opposing auxin flows (indicated by the yellow arrows). The sharp AD and AB boundary marked by a black line is located near the apical surface of the incipient primordium and responsible for the upward growth of the hollow tube. Mutants of the auxin signaling component and abaxialization factor *ETT/ARF3* have compromised abaxial identity (Pekker et al., 2005), which may lead to partially adaxialized carpels and hence enlarged adaxial tissues like stigma and style. In weak *ett* mutants (**Figure 6B**), a compromised abaxial domain means a reduced AD/AB boundary at the time of carpel primordium emergence (approximately floral stages 3–4). This is indicated by a short black line (AD/AB boundary) at the apical surface of the incipient primordium (compare **Figure 6Bi** with **Figure 6Ai**) and a shorter gynoecium tube (**Figure 6Bii**). In support of an early role of AD/AB polarity in specifying gynoecium patterning, double mutants of the *KAN* gene family with compromised abaxial identity also exhibit similar gynoecium phenotypes to *ett* mutants (Eshed et al., 2001; Pekker et al., 2005).

Mutants defective in auxin polar transport (in *pin* or *pid* mutants, or by NPA treatment) exhibit weakened or absent auxin flows into the incipient carpel primordium (**Figures 6Ci,iii**), which will lead to a lack of a clear AD/AB boundary in the incipient carpel primordium indicated by a lack of the black line. As a result no valve or a reduced valve will form. Mutants of auxin biosynthesis (in *yuc1 yuc4* or *taa/tar* mutants) likely have insufficient auxin to be transported toward the incipient primordium, resulting in the absence of AD/AB domains and hence a lack of gynoecium tube (**Figures 6Di,iii**).

In all auxin-pathway mutants (*yuc, taa/tar, pin, pid,* and *ett*), the severity of the defects caused by different alleles negatively correlates the extent to which an AD/AB boundary remains in the primordium. The stronger the defects, the smaller the AD/AB boundary is at the apex, and the smaller the valve. The resulting non-polarized zone at the base of the primordium may lead to a longer gynophore at the base. Gynophore elongation may be regulated by a separate growth mechanism that is related to the proximal–distal growth and independent of the AD/AB polarity.

This early action model cannot explain why the *yuc1 yuc4* or *pin,* or *pid* mutants are still capable of developing almost normal amount of stigmatic tissues at the apex, other than by proposing that the stigma development may occur later, after the apical to basal patterning of gynoecium is established. *STYLISH1/2* and *NGA3* transcription factors are known to activate the late-stage *YUC* gene expression required for stigma development (Sohlberg et al., 2006; Trigueros et al., 2009; Eklund et al., 2010). The fact that *yuc4 yuc1* double mutants still develop stigmatic tissues hints at additional redundancy in sources of auxin for the apex of the gynoecium. This redundancy could be caused by other *YUC* genes such as *YUC2,* which is expressed broadly in floral primordia (Cheng et al., 2006), or by upward transport of auxin via PIN1 localized to the replum cells (Grieneisen et al., 2013). As the replum represents the medial edge of the carpels, this pattern of upward transport is strikingly reminiscent of the Berleth et al. (2007) model of auxin’s movement in aerial organs discussed earlier, which has auxin from the stem being transported up the leaf along its medial edges.

This early action model could be evaluated experimentally by looking at the expression of genes in the AD/AB cohorts at very early stages of gynoecial development. Under this model, we would expect that *pin1*, *pid*, or *yuc1 yuc4* double mutants fail to show a clear AD/AB boundary in carpel primordia and that *ett* mutants express expanded adaxial-specific molecular markers and shrinking abaxial-specific markers due to adaxialization of carpels. In contrast, the Nemhauser apical gradient model does not imply such a result.

## CONCLUSION

Fourteen years ago, Nemhauser et al. (2000) proposed the auxin gradient model to explain the apical-to-basal morphogenesis of the *Arabidopsis gynoecium*. While it is a highly attractive model, the auxin gradient, on which the Nemhauser model heavily relies, remains elusive and multiple observations made since are inconsistent with aspects of the model. Here, we have proposed an alternative model, the early action model, based on three observations. One is the timing of the apical-to-basal patterning, which occurs much earlier than the observed auxin biosynthesis at the gynoecium apex. Another is the already-established evolutionary homology between carpel and leaf-like lateral organs. The third is the set of emerging models of auxin’s role in leaf and lateral organ development, including the link between auxin transport, synthesis, and signaling and lateral organs’ AD/AB boundary establishment. Our model emphasizes auxin’s early effects on AD/AB boundary establishment as an explanation for the defects of gynoecium in apical–basal patterning induced by auxin-disrupting mutations and chemicals. Furthermore, the early action model unifies the development of carpels with current models of the development of other lateral organs.

## Conflict of Interest Statement

The authors declare that the research was conducted in the absence of any commercial or financial relationships that could be construed as a potential conflict of interest.
